# The Impact of Person-Centered Communication Education on Nursing Home Regulatory Survey Outcomes

**DOI:** 10.1093/geroni/igaf122.2697

**Published:** 2025-12-31

**Authors:** Kristine Williams, Clarissa Shaw, Shelby Cooper, Maria Hein, Yelena Perkhounkova, Carissa Coleman, Frances Yang, Maria Roche-Dean

**Affiliations:** University of Kansas, Kansas City, Kansas, United States; University of Iowa College of Nursing, Iowa City, Iowa, United States; University of Kansas, Lawrence, Kansas, United States; University of Iowa, Iowa City, Iowa, United States; University of Iowa, Iowa City, Iowa, United States; University of Kansas Medical Center, Kansas City, Kansas, United States; University of Kansas, University of Kansas Medical Center, Kansas, United States; Kirkhof College of Nursing, Grand rapids, Michigan, United States

## Abstract

Elderspeak is a form of infantilizing communication commonly used with older adults in nursing homes. This study evaluated the impact of the Changing Talk Online training (CHATO) on standard nursing home annual health inspector surveys in a multi-state pragmatic clinical trial. CHATO is an asynchronous online intervention educating nursing home staff person-centered communication skills through the reduction of elderspeak. Annual health survey citations were examined for 14 F-tag deficiency categories related to resident rights, quality of care, and quality assurance one year before and one year after study participation. Sixty-six of the 142 participating nursing homes had health standard inspections both before and after the study period (intervention n = 31, control n = 35.) Prior to the study period, nine control nursing homes received 13 citations, and six intervention nursing homes received seven citations, which was not significantly different at the nursing home level (Fisher’s exact test, p=.770). Following the study period, 11 control nursing homes received 14 citations, and two intervention nursing homes received three citations, which is significantly different at the nursing home level (Fisher’s exact test, p = 0.015). These preliminary findings suggest that CHATO communication training may help nursing homes focus on improving person-centered care and reduce related health inspection deficiencies. While the trial’s primary outcomes focus on resident-centered measures from Minimum Data Set (MDS) data that directly reflect the resident’s well-being, these deficiency findings may be particularly compelling for nursing home corporations and owners given their connection to financial penalties and regulatory compliance.

